# Supplementary material for: USP17L promotes the 2-cell-like program through deubiquitination of H2AK119ub1 and ZSCAN4
Source: Nat Commun. 2025 Aug 1;16:7071. doi: 10.1038/s41467-025-62303-x (PMC12316976; doi:10.1038/s41467-025-62303-x)
Supplement: Supplementary file 1 — Supplementary Information [file 41467_2025_62303_MOESM1_ESM.pdf]

Supplementary figures and tables

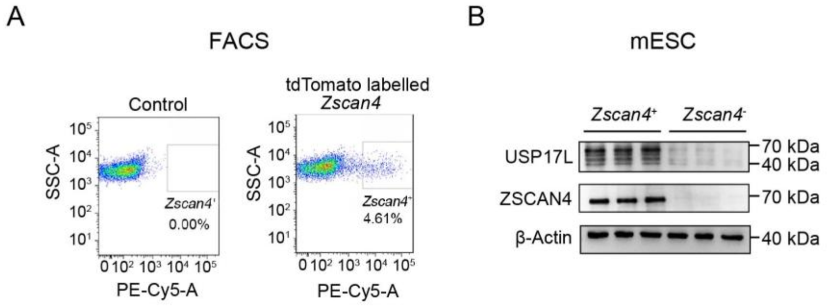

Supplementary Fig. 1 | USP17L is highly expressed in *Zscan4*$^+$ ESCs. A Flow cytometry sorting of *Zscan4*$^+$ and *Zscan4*$^−$ ESCs based on tdTomato in control and tdTomato-labelled ZSCAN4 expressing ESCs. **B** Western blot showing the expression of USP17L protein in *Zscan4*$^+$ and *Zscan4*$^−$ ESCs. β-Actin was used as loading control (3 biological replicates).

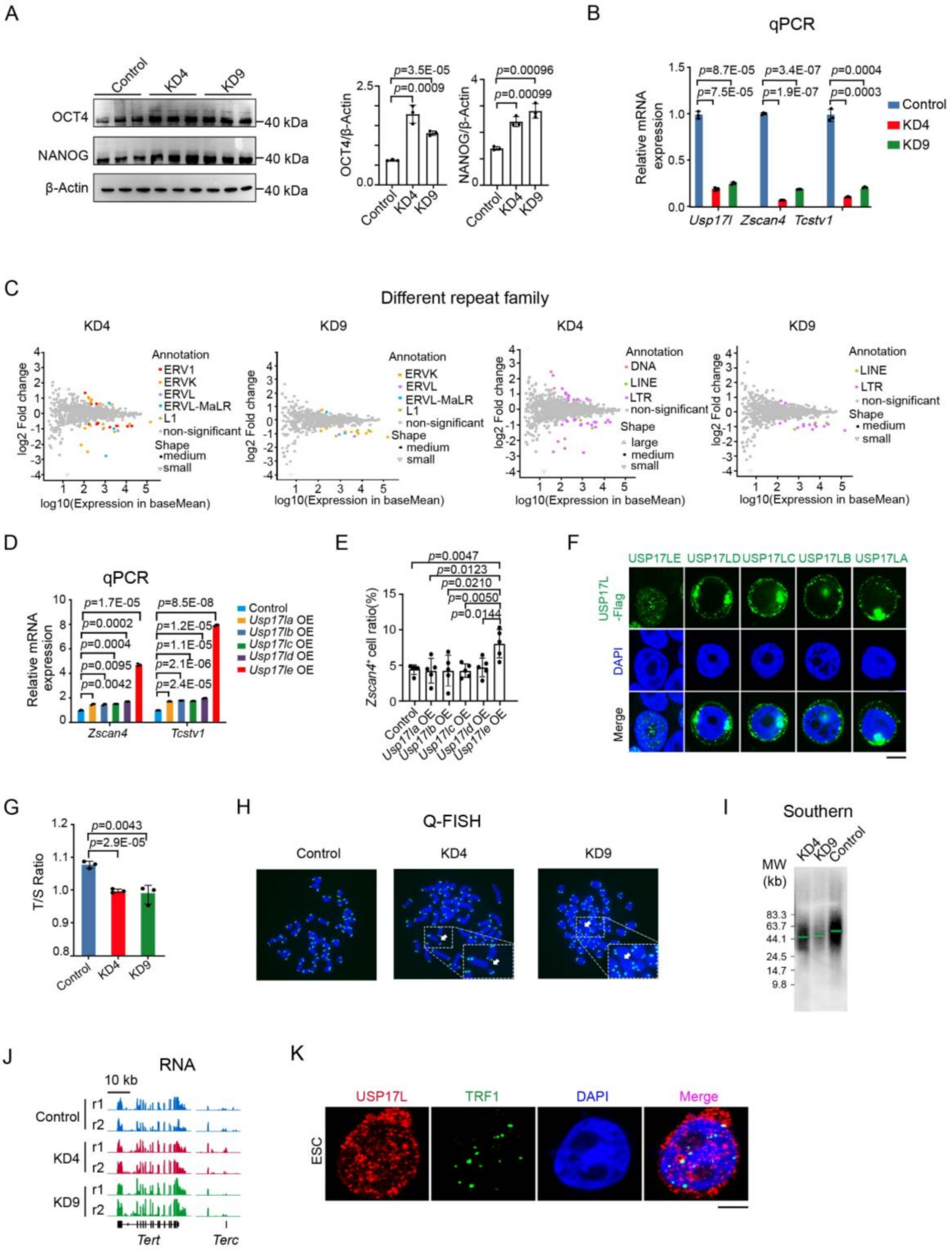

Supplementary Fig. 2 | *Usp17l* regulates 2C genes and telomere. A Western blot showing the changes of OCT4 and NANOG in *Usp17l* knockdown ESCs. The relative levels of OCT4 and NANOG were determined by densitometry of the protein bands (3 biological replicates). Data were shown as means ± SD (two-tailed Student's t-test). **B** RT-qPCR analysis showing the relative expression levels of *Usp17l*, *Zscan4*, and *Tcstv1* in

control and *Usp17l* knockdown ESCs (3 biological replicates). Data were shown as means ± SD (two-tailed Student's t-test). **C** Scatter plot showing the expression of different family of repeats after *Usp17l* knockdown. **D** RT-qPCR analysis showing the relative expression levels of *Zscan4* and *Tcstv1* in control and individual *Usp17l* overexpressing ESCs (3 biological replicates). Data were shown as means ± SD (two-tailed Student's t-test). **E** Statistics of the ratios of *Zscan4*$^{+}$ cells following transient overexpression of *Usp17l* family genes for 48 h (3 biological replicates). Data were shown as means ± SD (two-tailed Student's t-test). **F** Immunofluorescence staining using anti-Flag antibody showing the localization of USP17L proteins in ESCs. Scale bar, 5 μm. DNA is stained with DAPI. **G** qPCR analysis showing telomere length changes following knockdown of *Usp17l* (3 biological replicates). Data were shown as means ± SD (two-tailed Student's t-test). **H** Immunofluorescence staining showing the length of telomeres by Q-FISH. Arrows indicate chromosome breakage. **I** Telomere restriction fragment (TRF) measured by Southern blot showing the telomere length in control and *Usp17l* knockdown ESCs. Green bars indicate the average telomere length. **J** IGV visualization showing the RNA signals of *Tert* and *Terc* after *Usp17l* knockdown in ESCs. Two replicates are shown. **K** Co-immunostaining and fluorescence microscopy for USP17L (red) and TRF1 (green) in ESCs. DNA is stained with DAPI. Scale bar, 5 μm.

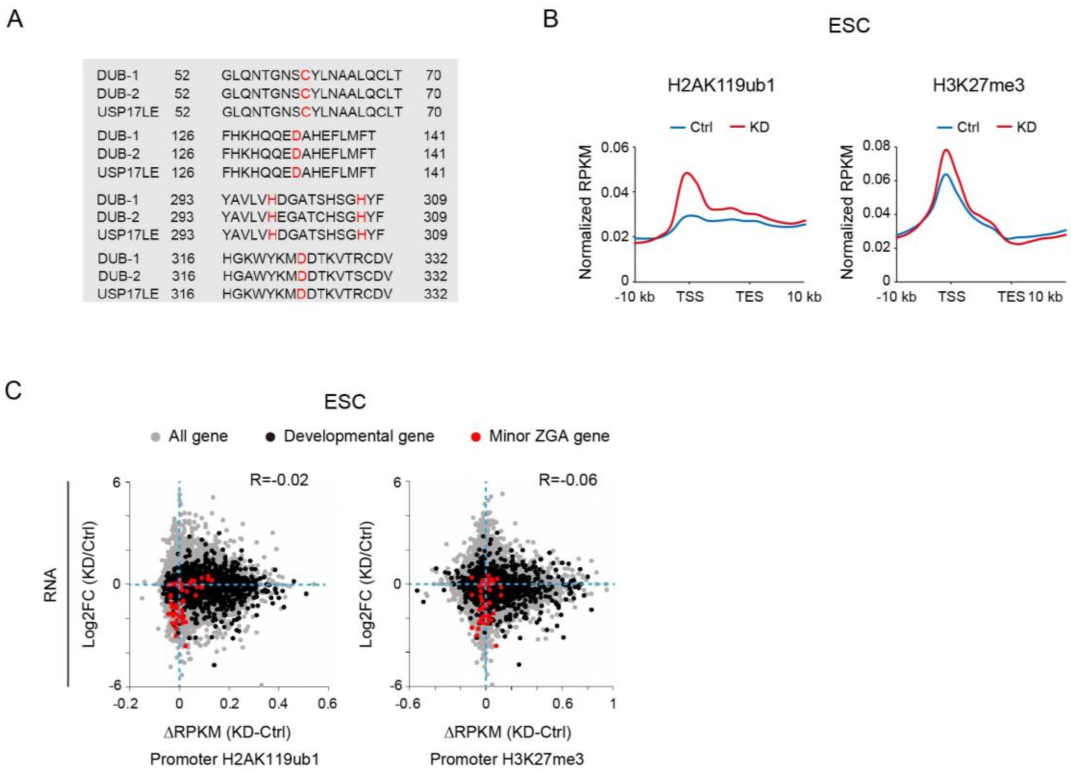

Supplementary Fig. 3 | The effect of *Usp17l* KD on H2AK119ub1, H3K27me3 and gene expression in ESCs. **A** The conserved domain and catalytic sites (red) of USP17LE revealed by amino acid alignment. These amino acid sequences were derived from[1]. **B** Line charts showing the global H2AK119ub1 (left) and H3K27me3 (right) in control and *Usp17l* KD mESCs. **C** Scatter plots comparing gene expression changes and the changes of H2AK119ub1 (left) and H3K27me3 (right) at gene promoters. Developmental genes and minor ZGA genes are highlighted in black and red, respectively.

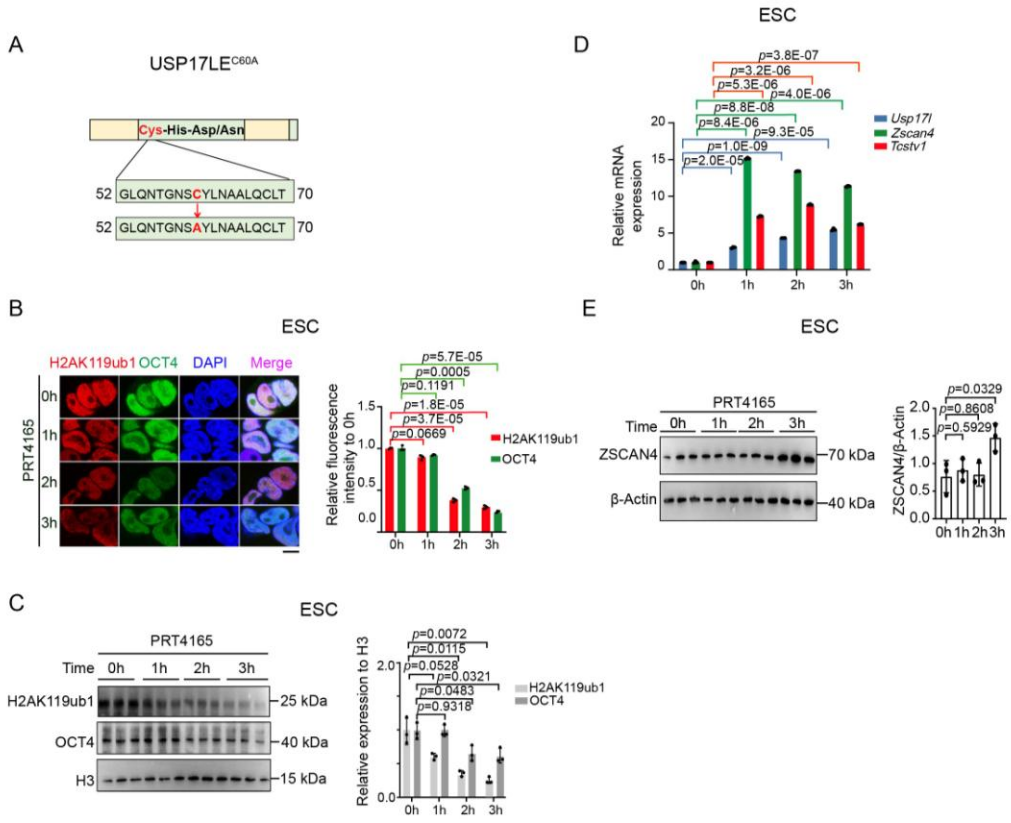

Supplementary Fig. 4 | H2AK119ub1 regulates the expression of 2C-genes. A Schematic showing the mutation of the conserved catalytic site (C60A) in USP17LE. **B** Left, immunofluorescence staining for H2AK119ub1 (red) and OCT4 (green) in ESCs following inhibition of H2AK119ub1 by a specific inhibitor PRT4165 (50 μM) for indicated time. DNA is stained with DAPI. Scale bar, 5 μm. Right, statistics of the relative fluorescence intensity of H2AK119ub1 and OCT4 after treatment of PRT4165 (3 biological replicates). Data were shown as means ± SD (two-tailed Student's t-test). **C** Left, western blot showing the changes of H2AK119ub1 and OCT4 in ESCs following inhibition of H2AK119ub1 by PRT4165 (50 μM) for indicated time. H3 is used as loading control. Right, the relative levels of H2AK119ub1 and OCT4 determined by densitometry of the protein bands (3 biological replicates). Data were shown as means ± SD (two-tailed Student's t-test). **D** RT-qPCR analysis showing the relative expression level of *Usp17l*, *Zscan4* and *Tcstv1* after inhibition of H2AK119ub1 for indicated time (3 biological replicates). Data were shown as means ± SD (two-tailed Student's t-test). **E** Left, western blot showing the levels of ZSCAN4 protein following inhibition of H2AK119ub1 by PRT4165 in E3 ESCs for indicated time. Right, the relative level of ZSCAN4

protein determined by densitometry of the protein bands (3 biological replicates). Data were shown as means ± SD (two-tailed Student’s t-test).

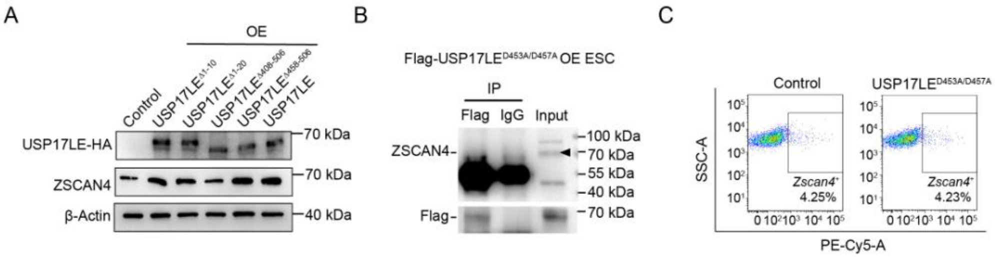

Supplementary Fig. 5 | USP17LE stabilizes ZSCAN4 through deubiquitination. **A** Western blot showing the changes of ZSCAN4 protein levels after overexpression of various truncated mutants of USP17LE (2 biological replicates). **B** Western blot showing the loss of interaction between ZSCAN4 and USP17LE$^{D453/D457}$. Co-IP was performed using anti-Flag antibody in USP17LE$^{D453/D457}$ overexpressing ESCs. IgG serves as control. The black arrow head indicates the position of ZSCAN4 (3 biological replicates). **C** Flow cytometry analysis of *Zscan4*$^{+}$ cells in control and USP17LE$^{D453/D457}$ overexpressing *Zscan4*::tdTomato ESCs.

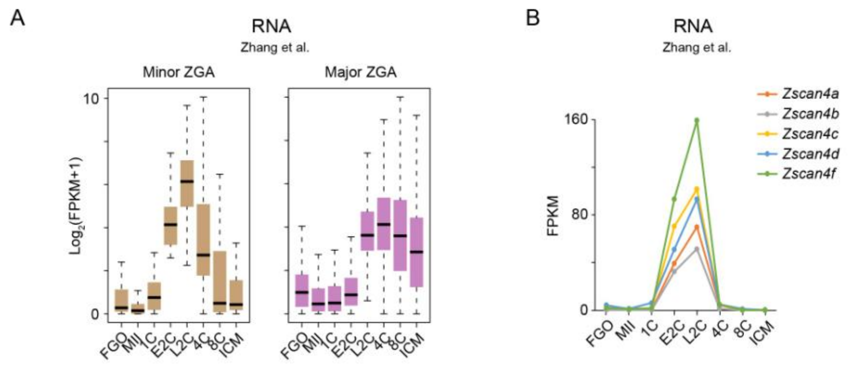

Supplementary Fig. 6 | The expression of minor and major ZGA genes during mouse pre-implantation development. A Bar chart showing the expression of minor (left) and major ZGA genes (right) in WT embryos during mouse pre-implantation development. FGO, Full-grown GV oocyte; MII, metaphase II oocyte; 1C, 1-cell; E2C, early 2-cell; L2C, late 2-cell; 4C, 4-cell; 8C, 8-cell; ICM, inner cell mass. RNA-seq data are previously published[2]. **B** Line chart showing the expression of *Zscan4* family genes during mouse pre-implantation development. RNA-seq data are previously published[2].

Supplemental Tables

Supplementary Table 1 Primers for RT-qPCR.

Usp17la-F<br>*Usp17la*-R	AGAAGATTTAGGAGAGCCCTGCG<br>TCCTGAAGCACTTTCCCCTTTC
Usp17lb-F<br>*Usp17lb*-R	GTGGTTGCTCTCTCCTTCCC<br>TCCTCTACCACCTGGGCTTC
Usp17lc-F<br>*Usp17lc*-R	CAAAGCCAGACATGGGGCAT<br>GCACATAGGCATTCTCATTCAGG
Usp17ld-F<br>*Usp17ld*-R	CCTGCCCAGATCCATAGCAA<br>GTGAGGAGCCTGTCAGCATT
Usp17le-F<br>*Usp17le*-R	TCCTTCCCAGAAGAGACTGGA<br>AGCAACCACCATGTCTCCAA
Zscan4-F<br>*Zscan4*-R	AAATGCCTTATGTCTGTTCCCTATG<br>TGTGGTAATTCCTCAGGTGACGAT
Tcstv1-F<br>*Tcstv1*-R	TGAACCCTGATGCCTGCTAAGACT<br>AGATGGCTGCAAAGACACAACTGC
Tcstv3-F<br>*Tcstv3*-R	AGAAAGGGCTGGAACTTGTGACCT<br>AAAGCTCTTTGAAGCCATGCCCAG

Supplementary Table 2 Primers for gene overexpression experiments.

Usp17la -Nhe1-F<br>*Usp17la* -Not1-R	CTAGCTAGCATGGTGGTTGCTCTTTCCTTCCC<br>AAGGAAAAAAGCGGCCGCCTAGCAAACAAGCAGAAGCCTCTGC
Usp17lb -Nhe1-F<br>*Usp17lb* -Not1-R	CTAGCTAGCATGGTGGTTGCTCTCTCCTTCCC<br>AAGGAAAAAAGCGGCCGCTCAGTTTGCAGTGCATATAGGTTGG
Usp17lc -Nhe1-F<br>*Usp17lc* -Not1-R	CTAGCTAGCATGGTGGTTTCTCTTTCCTTCCC<br>AAGGAAAAAAGCGGCCGCCTAGCAAACAAGCAGAAGCCTCTGC
Usp17ld -Nhe1-F<br>*Usp17ld* -Not1-R	CTAGCTAGCATGGTGGTTTCTCTTTCCTTCCC<br>AAGGAAAAAAGCGGCCGCCTAGCGAACAAGCAGAAGCTTCTGC

Usp17le -Nhe1-F<br>*Usp17le* -Not1-R	CTAGCTAGCATGGTGGTTTCTCTTTCCTTCCC<br>AAGGAAAAAAGCGGCCGCTCATCTCCCACCCTGACTACAGAGC
Usp17le-IVT-F<br>*Usp17le*-IVT-R	TAATACGACTCACTATAGGGAGAATGGTGGTTTCTCTTTCCTTC<br>CC<br>TCATCTCCCACCCTGACTACAGAGC

Supplementary Table 3 Primers for *Usp17l* mutation experiments.

USP17LE$^{D453/457}$-F<br>USP17LE$^{D453/457}$-R	TGCGGGCTCCAGGCCACAGGCAACAGCGCCTACCTG<br>AACGCAGCCCTGCA<br>CGTTCAGGTAGGCGCTGTTGCCTGTGGCCTGGAGCC<br>CGCATCCTGGTCCT
USP17LE$^{\Delta 1-10}$-Nhe1-F<br>USP17LEΔ$^{1-10}$-Not1-R	CTAGCTAGCATGGAGCTGCATCAGGATGAAGC<br>AAGGAAAAAAGCGGCCGCTCATCTCCCACCCTGACT<br>ACAGAGC
USP17LE$^{\Delta 1-20}$-Nhe1-F<br>USP17LE$^{\Delta 1-20}$-Not1-R	CTAGCTAGCATGCTGAGTTGGGAGAGTCCCCA<br>AAGGAAAAAAGCGGCCGCTCATCTCCCACCCTGACT<br>ACAGAGC
USP17LE$^{\Delta 407-506}$-Nhe1-F<br>USP17LE$^{\Delta 407-506}$-Not1-R	CTAGCTAGCATGGTGGTTTCTCTTTCCTTCCC<br>ATAAGAATGCGGCCGCCTTCTCCCTGTTTTCGCATG
USP17LE$^{\Delta 458-506}$-Nhe1-F<br>USP17LE$^{\Delta 458-506}$-Not1-R	CTAGCTAGCATGGTGGTTTCTCTTTCCTTCCC<br>ATAAGAATGCGGCCGCCACAATTGCATCAACAGGCA

Supplementary Table 4 Primers for combining pA-Tn5 adapter.

Primer A	5'-phos-CTGTCTCTTATACACATCT-NH2-3'
Primer B	5′-TCGTCGGCAGCGTCAGATGTGTATAAGAGACAG-3′
Primer C	5′-GTCTCGTGGGCTCGGAGATGTGTATAAGAGACAG-3′

Supplementary References

1. Shin JM, Yoo KJ, Kim MS, Kim D, Baek KH. Hyaluronan- and RNA-binding deubiquitinating enzymes of USP17 family members associated with cell viability. *BMC Genomics* **7**, 292 (2006).
2. Zhang B*, et al.* Allelic reprogramming of the histone modification H3K4me3 in early mammalian development. *Nature* **537**, 553-557 (2016).
