# Supplementary material for: USP17L promotes the 2-cell-like program through deubiquitination of H2AK119ub1 and ZSCAN4
Source: Nat Commun. 2025 Aug 1;16:7071. doi: 10.1038/s41467-025-62303-x (PMC12316976; doi:10.1038/s41467-025-62303-x)
Supplement: Supplementary file 3 — Reporting Summary [file 41467_2025_62303_MOESM3_ESM.pdf]

nature portfolio

Corresponding author(s): Wei Xie, Lin Liu

Last updated by author(s): Jun 24, 2025

Reporting Summary

Nature Portfolio wishes to improve the reproducibility of the work that we publish. This form provides structure for consistency and transparency in reporting. For further information on Nature Portfolio policies, see our Editorial Policies and the Editorial Policy Checklist.

Statistics

For all statistical analyses, confirm that the following items are present in the figure legend, table legend, main text, or Methods section.

n/a	Confirmed	
☐	☒	The exact sample size (*n*) for each experimental group/condition, given as a discrete number and unit of measurement
☐	☒	A statement on whether measurements were taken from distinct samples or whether the same sample was measured repeatedly
☐	☒	The statistical test(s) used AND whether they are one- or two-sided<br>*Only common tests should be described solely by name; describe more complex techniques in the Methods section.*
☐	☒	A description of all covariates tested
☐	☒	A description of any assumptions or corrections, such as tests of normality and adjustment for multiple comparisons
☐	☒	A full description of the statistical parameters including central tendency (e.g. means) or other basic estimates (e.g. regression coefficient) AND variation (e.g. standard deviation) or associated estimates of uncertainty (e.g. confidence intervals)
☐	☒	For null hypothesis testing, the test statistic (e.g. *F*, *t*, *r*) with confidence intervals, effect sizes, degrees of freedom and *P* value noted<br>*Give P values as exact values whenever suitable.*
☒	☐	For Bayesian analysis, information on the choice of priors and Markov chain Monte Carlo settings
☒	☐	For hierarchical and complex designs, identification of the appropriate level for tests and full reporting of outcomes
☐	☒	Estimates of effect sizes (e.g. Cohen's *d*, Pearson's *r*), indicating how they were calculated

Our web collection on statistics for biologists contains articles on many of the points above.

Software and code

Policy information about availability of computer code

Data collection	Public datasets were used in this paper as described and cited in the figure legend section.<br>Sequencing was performed using Illumina Hi-seq XTen or DNBSEQ-T7 platform.<br>Immunofluorescence were acquired by Zeiss Axio Imager Z1 or Zeiss LSM710.
Data analysis	No custom software was used in this study.<br>Sequencing data were analyzed as described in the Methods section. Tools and softwares used in this study are as follows:<br><br>bowtie2:https://bowtie-bio.sourceforge.net/bowtie2<br>SAMtools:https://sourceforge.net/projects/samtools<br>deeptools:https://deeptools.readthedocs.io/en/develop/<br>IGVbrowser:https://igv.org<br>Hisat2:http://daehwankimlab.github.io/hisat2/<br>Featurecounts:https://subread.sourceforge.net/featureCounts.html<br>DEseq2:https://bioconductor.org/packages/release/bioc/html/DESeq2.html<br>Genome:ftp://hgdownload.soe.ucsc.edu/goldenPath/mm10/

For manuscripts utilizing custom algorithms or software that are central to the research but not yet described in published literature, software must be made available to editors and reviewers. We strongly encourage code deposition in a community repository (e.g. GitHub). See the Nature Portfolio guidelines for submitting code & software for further information.

Data

Policy information about availability of data

All manuscripts must include a data availability statement. This statement should provide the following information, where applicable:

- Accession codes, unique identifiers, or web links for publicly available datasets
- A description of any restrictions on data availability
- For clinical datasets or third party data, please ensure that the statement adheres to our policy

All sequencing data generated in this study have been deposited to NCBI GEO and are publicly available. The accession numbers for RNA-seq data are GSE223067 (Usp17l KD mESCs), GSE234366 (Usp17le OE mESCs), GSE277982 (WT/Dux KD/Dux KD-Usp17le OE/Usp17le OE/ Usp17le OE-Dux KD mESCs), and GSE282420 (Usp17l KD and Usp17le OE late 2C embryos). The accession number for CUT&Tag data is GSE224711. The accession number for STAR ChIP-seq, Stacc-seq, and CUT&RUN data is GSE282421. Source data are provided with this paper.

Research involving human participants, their data, or biological material

Policy information about studies with human participants or human data. See also policy information about sex, gender (identity/presentation), and sexual orientation and race, ethnicity and racism.

Reporting on sex and gender	None
Reporting on race, ethnicity, or other socially relevant groupings	None
Population characteristics	None
Recruitment	None
Ethics oversight	None

Note that full information on the approval of the study protocol must also be provided in the manuscript.

Field-specific reporting

Please select the one below that is the best fit for your research. If you are not sure, read the appropriate sections before making your selection.

[x] Life sciences [ ] Behavioural & social sciences [ ] Ecological, evolutionary & environmental sciences

For a reference copy of the document with all sections, see nature.com/documents/nr-reporting-summary-flat.pdf

Life sciences study design

All studies must disclose on these points even when the disclosure is negative.

Sample size	No statistical methods were used to predetermine sample sizes.
Data exclusions	No data was excluded from the analysis.
Replication	Experimental findings were reliably reproduced. Experimental replication was attempted for all datasets/figures shown.
Randomization	Experimental materials were not divided into random subgroups. Most comparisons were done between WT and mutants.
Blinding	Not applicable since there was no specific grouping.

Reporting for specific materials, systems and methods

We require information from authors about some types of materials, experimental systems and methods used in many studies. Here, indicate whether each material, system or method listed is relevant to your study. If you are not sure if a list item applies to your research, read the appropriate section before selecting a response.

Materials & experimental systems

n/a	Involved in the study
☐	☒ Antibodies
☐	☒ Eukaryotic cell lines
☒	☐ Palaeontology and archaeology
☐	☒ Animals and other organisms
☒	☐ Clinical data
☒	☐ Dual use research of concern
☒	☐ Plants

Methods

n/a	Involved in the study
☐	☒ ChIP-seq
☐	☒ Flow cytometry
☒	☐ MRI-based neuroimaging

Antibodies

Antibodies used

H3K9me2:Abcam (ab1220); Nanog: Abcam (ab80892); H3K27me3: Millipore (07-449); H3K27me3: CST (9733s); H3K9me3: Abcam (ab8898); H3K9me3: Active motif (39161); H4K20me3: Millipore (07-463-S); H2AK119ub1: CST(8240S); Zscan4: Millipore (AB4340); Oct4: Santa Cruz (sc-5279); β-Actin: Abclonal (AC026); Anti-Ubiquitin: Abcam (ab140601); Flag: SIGMA (F1804-50UG); AlexaFluor® 594 Goat Anti-Rabbit IgG: Life (A-11037); AlexaFluor® 594 Goat Anti-Mouse IgG: Life (A-11005); AlexaFluor® 594 Donkey anti-Goat IgG: Life (A-11058); AlexaFluor® 488 Donkey anti-Rabbit IgG: Life (A-21206); AlexaFluor® 488 Donkey anti-Mouse IgG: Life (A-21202); FITC Goat anti-Mouse IgG (H+L): Jackson (115-095-003).TRF1: Abcam(ab192629-100ug)

Validation

All the antibodies used in this study were commercial antibodies and were only used for applications, with validation procedures described on the following sites of the manufacturers and relevant citations:
Rabbit anti-H2AK119ub1: https://www.cellsignal.com/products/primary-antibodies/ubiquityl-histone-h2a-lys119-d27c4-xp-rabbit-mab/8240
Mouse anti-H3K27me3: https://www.cellsignal.cn/products/primary-antibodies/tri-methyl-histone-h3-lys27-c36b11-rabbit-mab/9733
mouse anti-FLAG: https://www.sigmaaldrich.cn/CN/zh/product/sigma/f1804
mouse anti-OCT4: https://www.scbt.com/p/oct-3-4-antibody-c-10
rabbit anti-HA: extension://idghocbbahafpfhjnfhpbfbmpegphmmp/assets/pdf/web/viewer.html?file=https%3A%2F%2Fstream.yeasen.com%2Fyshop%2F6391b1899932e9f46b8590df.pdf
rabbit anti-ACTIN: extension://idghocbbahafpfhjnfhpbfbmpegphmmp/assets/pdf/web/viewer.html?file=https%3A%2F%2Fabclonal.com.cn%2FDatasheet%2FAntibodies%2FAC026.pdf%3Fv%3D1703639961
rabbit anti-H3K9me2: https://www.abcam.cn/products/primary-antibodies/histone-h3-di-methyl-k9-antibody-mabcam-1220-chip-grade-ab1220.html
rabbit anti-H3K9me3: https://www.abcam.cn/products/primary-antibodies/histone-h3-tri-methyl-k9-antibody-chip-grade-ab8898.html
rabbit anti- H3K27me3: https://www.sigmaaldrich.cn/CN/zh/product/mm/07449
rabbit anti-H3K4me: https://www.abcam.cn/products/primary-antibodies/histone-h3-mono-methyl-k4-antibody-erp16597-chip-grade-ab176877.html
rabbit anti-H4K20me3: https://www.sigmaaldrich.cn/CN/zh/product/mm/07463s
rabbit anti-Nanog: https://www.abcam.cn/products/primary-antibodies/nanog-antibody-ab80892.html
goat anti-mouse HRP: https://www.abways.cn/ProductsStd/AB0102.html
goat anti-rabbit HRP: https://www.abways.com/ProductsStd/AB0101.html
Rabbit anti-ZSCAN: https://www.merckmillipore.com/CN/zh/product/Anti-Zscan4-Antibody,MM_NF-AB4340

Eukaryotic cell lines

Policy information about cell lines and Sex and Gender in Research

Cell line source(s)

All cell lines were established in our lab and information provided in Methods section.

Authentication

Pluripotency and naïve markers were expressed in our mouse mES cells validated by RNA-seq.

Mycoplasma contamination

All cell lines tested negative for mycoplasma contamination

Commonly misidentified lines
(See ICLAC register)

None

Animals and other research organisms

Policy information about studies involving animals; ARRIVE guidelines recommended for reporting animal research, and Sex and Gender in Research

Laboratory animals

WT C57BL/6J female mice (4 weeks), from Vital River;
PWK/PhJ males (6-12 weeks), from Jackson Laboratory.
All mice were housed in a Specific Pathogen Free (SPF) facility with individually ventilated cages. The room has controlled temperature (20-22°C), humidity (30%–70%) and light (12 hour light-dark cycle). Mice were provided ad libitum access to a regular

	rodent chow diet. All animal maintenance and experimental procedures used in current study were carried out according to guidelines of Institutional Animal Care and Use Committee (IACUC) of Tsinghua University, Beijing, China.
Wild animals	This study did not involve wild animals.
Reporting on sex	Sex was not considered in experiment design.
Field-collected samples	All animal maintenance and experimental procedures used in current study were carried out according to guidelines of Institutional Animal Care and Use Committee (IACUC) of Tsinghua University, Beijing, China.
Ethics oversight	All animal maintenance and experimental procedures were carried out according to the guidelines of the Institutional Animal Care and Use Committee (IACUC) of Tsinghua University, Beijing, China.

Note that full information on the approval of the study protocol must also be provided in the manuscript.

Plants

Seed stocks	None
Novel plant genotypes	None
Authentication	None

ChIP-seq

Data deposition

- [x] Confirm that both raw and final processed data have been deposited in a public database such as GEO.
- [ ] Confirm that you have deposited or provided access to graph files (e.g. BED files) for the called peaks.

Data access links
May remain private before publication.

GSE224711(secure token: idgrqqcmphkxvip)
https://www.ncbi.nlm.nih.gov/geo/query/acc.cgi?acc=GSE224711
GSE282421 (secure token: qzyjicasrdanbmp)
https://www.ncbi.nlm.nih.gov/geo/query/acc.cgi?acc=GSE282421

Files in database submission

Control-H2AK119ub _1_1.fastq.gz.gz
Control-H2AK119ub _1_2.fastq.gz.gz
Control-H2AK119ub _2_1.fastq.gz.gz
Control-H2AK119ub _2_2.fastq.gz.gz
KD9-H2AK119ub _1_1.fastq.gz.gz
KD9-H2AK119ub _1_2.fastq.gz.gz
KD9-H2AK119ub _2_1.fastq.gz.gz
KD9-H2AK119ub _2_2.fastq.gz.gz
Control-H3K27me3 _1_1.fastq.gz.gz
Control-H3K27me3 _1_2.fastq.gz.gz
KD9-H3K27me3 _1_1.fastq.gz.gz
KD9-H3K27me3 _1_2.fastq.gz.gz
KD9-H3K27me3 _2_1.fastq.gz.gz
KD9-H3K27me3 _2_2.fastq.gz.gz
Ctrl_L2C_H2AK119ub1_rep1_r1.fq.gz
Ctrl_L2C_H2AK119ub1_rep1_r2.fq.gz
Ctrl_L2C_H2AK119ub1_rep2_r1.fq.gz
Ctrl_L2C_H2AK119ub1_rep2_r2.fq.gz
Usp17le_OE_L2C_H2AK119ub1_rep1_r1.fq.gz
Usp17le_OE_L2C_H2AK119ub1_rep1_r2.fq.gz
Usp17le_OE_L2C_H2AK119ub1_rep2_r1.fq.gz
Usp17le_OE_L2C_H2AK119ub1_rep2_r2.fq.gz
Usp17l_KD_L2C_H2AK119ub1_rep1_r1.fq.gz
Usp17l_KD_L2C_H2AK119ub1_rep1_r2.fq.gz
Usp17l_KD_L2C_H2AK119ub1_rep2_r1.fq.gz
Usp17l_KD_L2C_H2AK119ub1_rep2_r2.fq.gz
Ctrl_L2C_H3K27me3_rep1_r1.fq.gz

Ctrl_L2C_H3K27me3_rep1_r2.fq.gz
Ctrl_L2C_H3K27me3_rep2_r1.fq.gz
Ctrl_L2C_H3K27me3_rep2_r2.fq.gz
Usp17le_OE_L2C_H3K27me3_rep1_r1.fq.gz
Usp17le_OE_L2C_H3K27me3_rep1_r2.fq.gz
Usp17le_OE_L2C_H3K27me3_rep2_r1.fq.gz
Usp17le_OE_L2C_H3K27me3_rep2_r2.fq.gz
Usp17l_KD_L2C_H3K27me3_rep1_r1.fq.gz
Usp17l_KD_L2C_H3K27me3_rep1_r2.fq.gz
Usp17l_KD_L2C_H3K27me3_rep2_r1.fq.gz
Usp17l_KD_L2C_H3K27me3_rep2_r2.fq.gz
WT_H3K9me3_rep1_r1.fq.gz
WT_H3K9me3_rep1_r2.fq.gz
WT_H3K9me3_rep2_r1.fq.gz
WT_H3K9me3_rep2_r2.fq.gz
Usp17l_KD4_H3K9me3_rep1_r1.fq.gz
Usp17l_KD4_H3K9me3_rep1_r2.fq.gz
Usp17l_KD4_H3K9me3_rep2_r1.fq.gz
Usp17l_KD4_H3K9me3_rep2_r2.fq.gz
Usp17l_KD9_H3K9me3_rep1_r1.fq.gz
Usp17l_KD9_H3K9me3_rep1_r2.fq.gz
Usp17l_KD9_H3K9me3_rep2_r1.fq.gz
Usp17l_KD9_H3K9me3_rep2_r2.fq.gz

Control_1_H2AK119ub.bw
Control_2_H2AK119ub.bw
KD9_1_H2AK119ub.bw
KD9_2_H2AK119ub.bw
Control_1_H3K27me3.bw
KD9_1_H3K27me3.bw
KD9_2_H3K27me3.bw
Ctrl_L2C_H2AK119ub1_rep1_reads_count.bw
Ctrl_L2C_H2AK119ub1_rep2_reads_count.bw
Usp17le_OE_L2C_H2AK119ub1_rep1_reads_count.bw
Usp17le_OE_L2C_H2AK119ub1_rep2_reads_count.bw
Usp17l_KD_L2C_H2AK119ub1_rep1_reads_count.bw
Usp17l_KD_L2C_H2AK119ub1_rep2_reads_count.bw
Ctrl_L2C_H3K27me3_rep1_reads_count.bw
Ctrl_L2C_H3K27me3_rep2_reads_count.bw
Usp17le_OE_L2C_H3K27me3_rep1_reads_count.bw
Usp17le_OE_L2C_H3K27me3_rep2_reads_count.bw
Usp17l_KD_L2C_H3K27me3_rep1_reads_count.bw
Usp17l_KD_L2C_H3K27me3_rep2_reads_count.bw
WT_H3K9me3_rep1_reads_count.bw
WT_H3K9me3_rep2_reads_count.bw
Usp17l_KD4_H3K9me3_rep1_reads_count.bw
Usp17l_KD4_H3K9me3_rep2_reads_count.bw
Usp17l_KD9_H3K9me3_rep1_reads_count.bw
Usp17l_KD9_H3K9me3_rep2_reads_count.bw

Genome browser session (e.g. UCSC)

None

Methodology

Replicates

Sample sizes for 2 biological replicates including all embryos and cell line CUT&Tag, STAR ChIP-seq,Stacc, and CUT&RUN in this study were used according to common practice in the field. The replicates were included in GEO accession GSE224711 and GSE282421.

Sequencing depth

Varies in different samples and can be checked at GEO accession

Antibodies

H3K9me3 (Active motif, 39161), H2AK119ub1 (CST, 8240S), H3K27me3 (Millipore, 07-449 for Cut&Tag; CST, 9733s for CUT&RUN)

Peak calling parameters

Our goal is to analysis the binding of the H2AK119ub/ H3K27me3 antibody on the set of genes without the call peaks step.

Data quality

Reads with a Phred quality score of<20 were removed. Non-unigue reads were removed by SAMtools. Quality were accessed bydeeptools and UCSC Genome Browser.

Software

Bowtie2 v2.3.5, samtools v1.3.1, deepTools v3.3.1

Flow Cytometry

Plots

Confirm that:

- [ ] The axis labels state the marker and fluorochrome used (e.g. CD4-FITC).
- [x] The axis scales are clearly visible. Include numbers along axes only for bottom left plot of group (a 'group' is an analysis of identical markers).
- [x] All plots are contour plots with outliers or pseudocolor plots.
- [x] A numerical value for number of cells or percentage (with statistics) is provided.

Methodology

Sample preparation	Information provided in Methods section. Cells prepared to perform Flow Cytomrtry are labled by Tdtomato. After being washed in PBS supplemented with 0.1% BSA, the cells were directly sorted and analyzed on a Flow Cytometer.
Instrument	BD Aria III
Software	BD FACSDiva acquisition foftware, FlowJo.
Cell population abundance	The abundance of the relevant cell populations, determined by testing the sorted cells again by FACS, reached >80%.
Gating strategy	Side-scatter area (SSC-A) versus forward-scatter area (FSC-A) and FSC-A versus forward-scatter width and profiles used to discard doublets and capture singlets.

- [ ] Tick this box to confirm that a figure exemplifying the gating strategy is provided in the Supplementary Information.
