# Supplementary material for: USP17L promotes the 2-cell-like program through deubiquitination of H2AK119ub1 and ZSCAN4
Source: Nat Commun. 2025 Aug 1;16:7071. doi: 10.1038/s41467-025-62303-x (PMC12316976; doi:10.1038/s41467-025-62303-x)

Figure 1F. USP17L was detected in USP17L knock-down cell lines

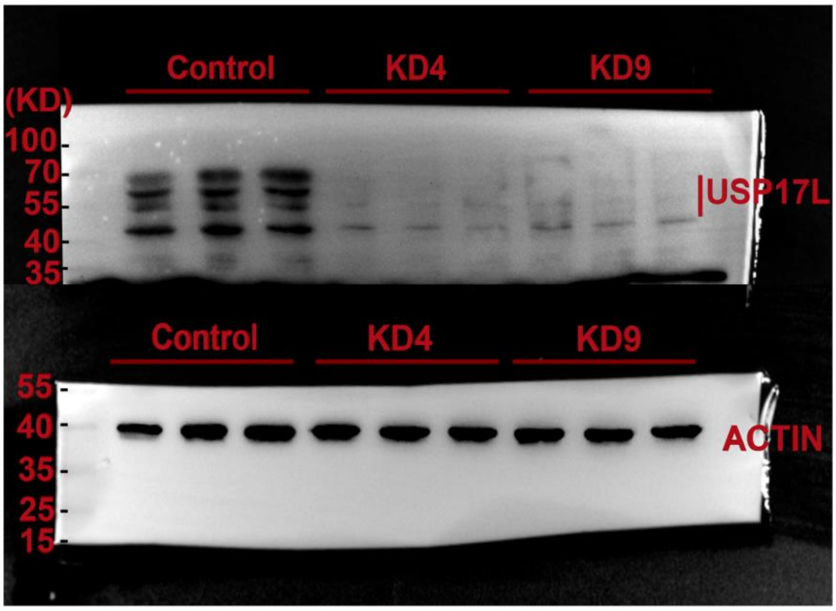

Figure 3A. Histone modifications were detected in USP17L knock-down cell lines

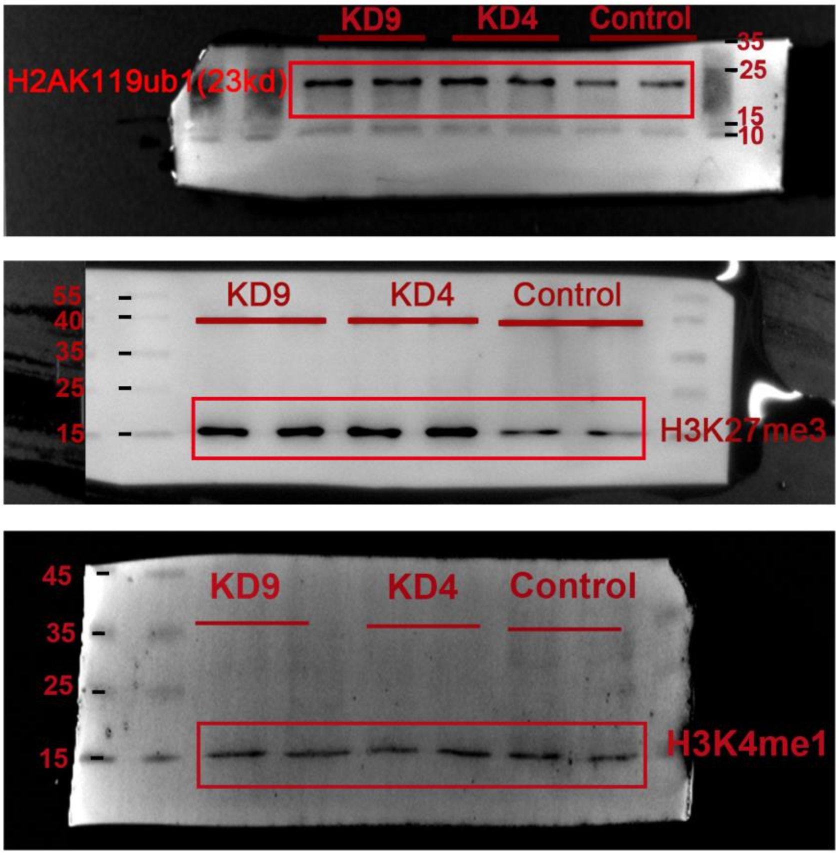

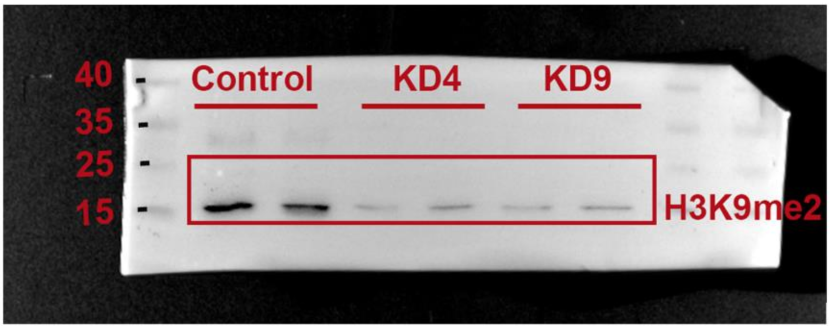
40
35
25
15
Control
KD4
KD9
H3K9me2

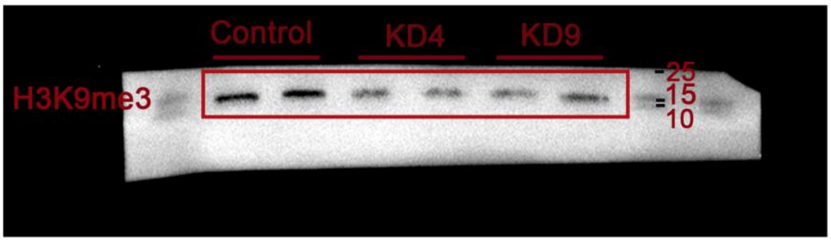
Control
KD4
KD9
H3K9me3
25
15
10

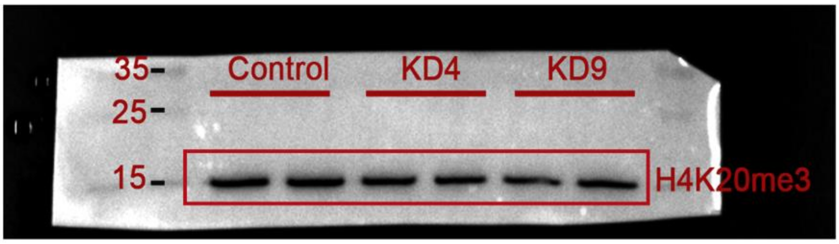
35
25
15
Control
KD4
KD9
H4K20me3

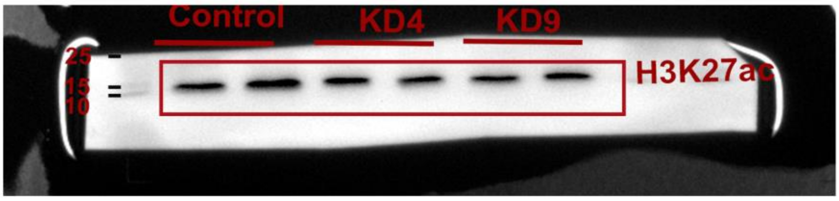
Control
KD4
KD9
25
15
10
H3K27ac

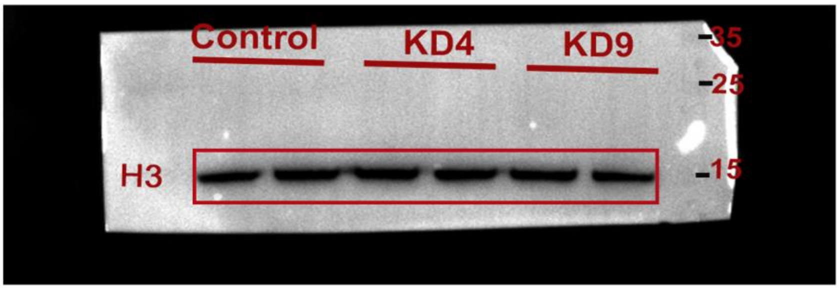
Control
KD4
KD9
35
25
15
H3

Figure 3B. USP17LE regulates H2AK119ub1 deubiquitination

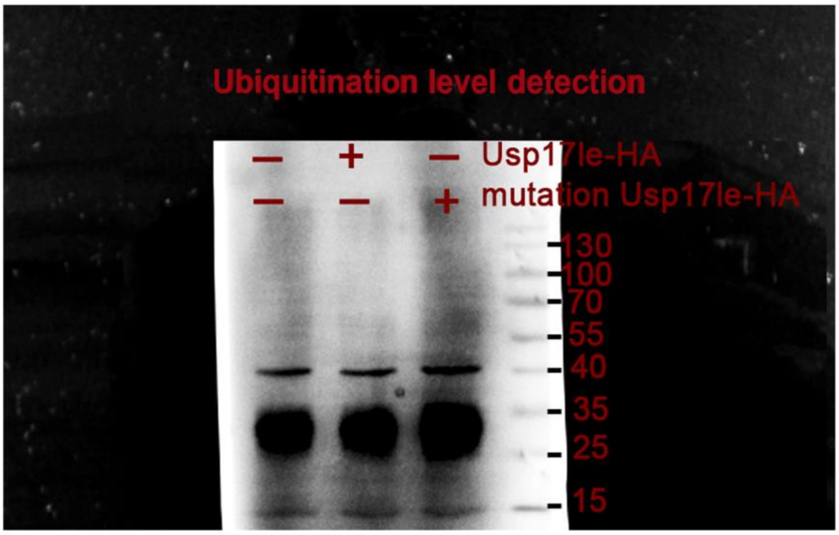

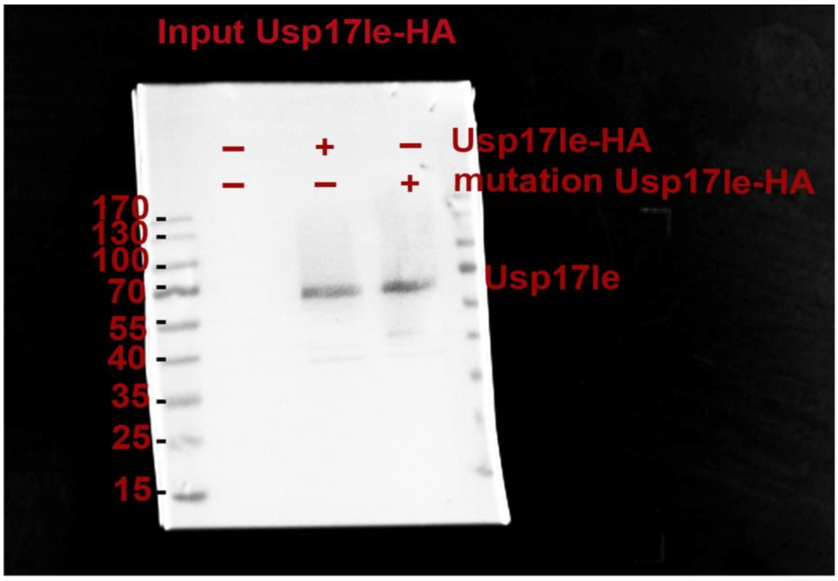

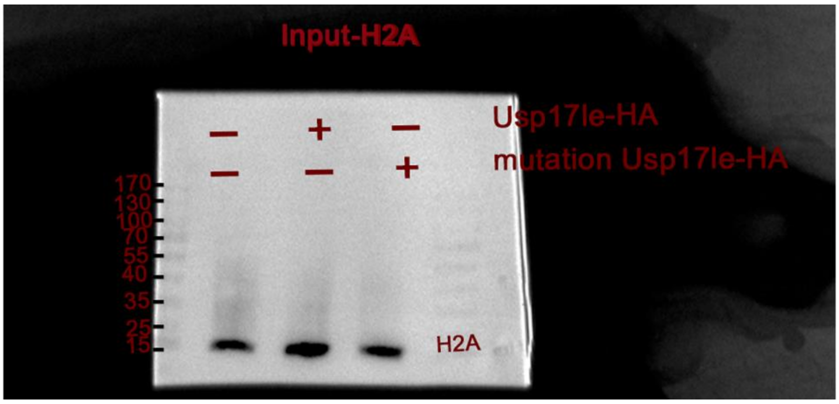

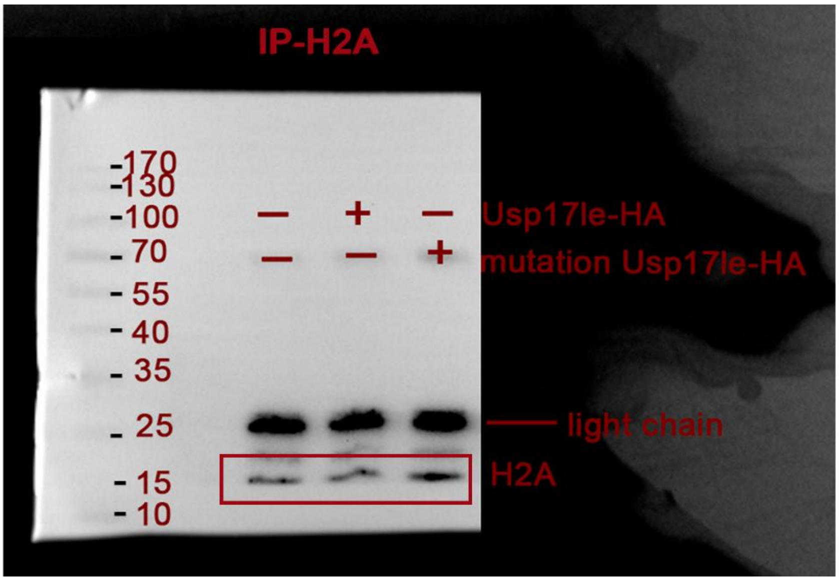
IP-H2A
170
130
100
70
55
40
35
25
15
10
Usp17le-HA
mutation Usp17le-HA
light chain
H2A

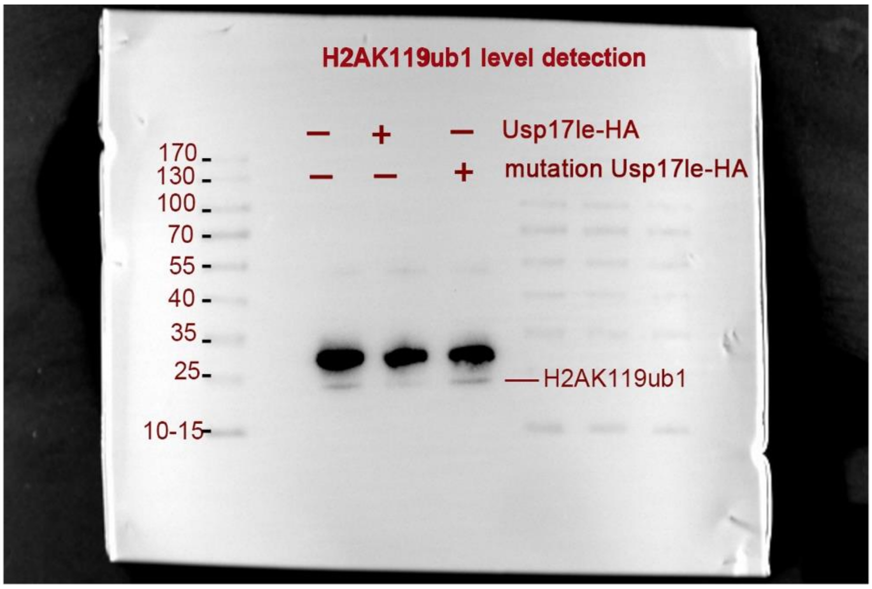
H2AK119ub1 level detection
Usp17le-HA
mutation Usp17le-HA
170
130
100
70
55
40
35
25
10-15
H2AK119ub1

Figure 3D. Overexpression of USP17LE can reduce H2AK119ub1 and H3K27me3

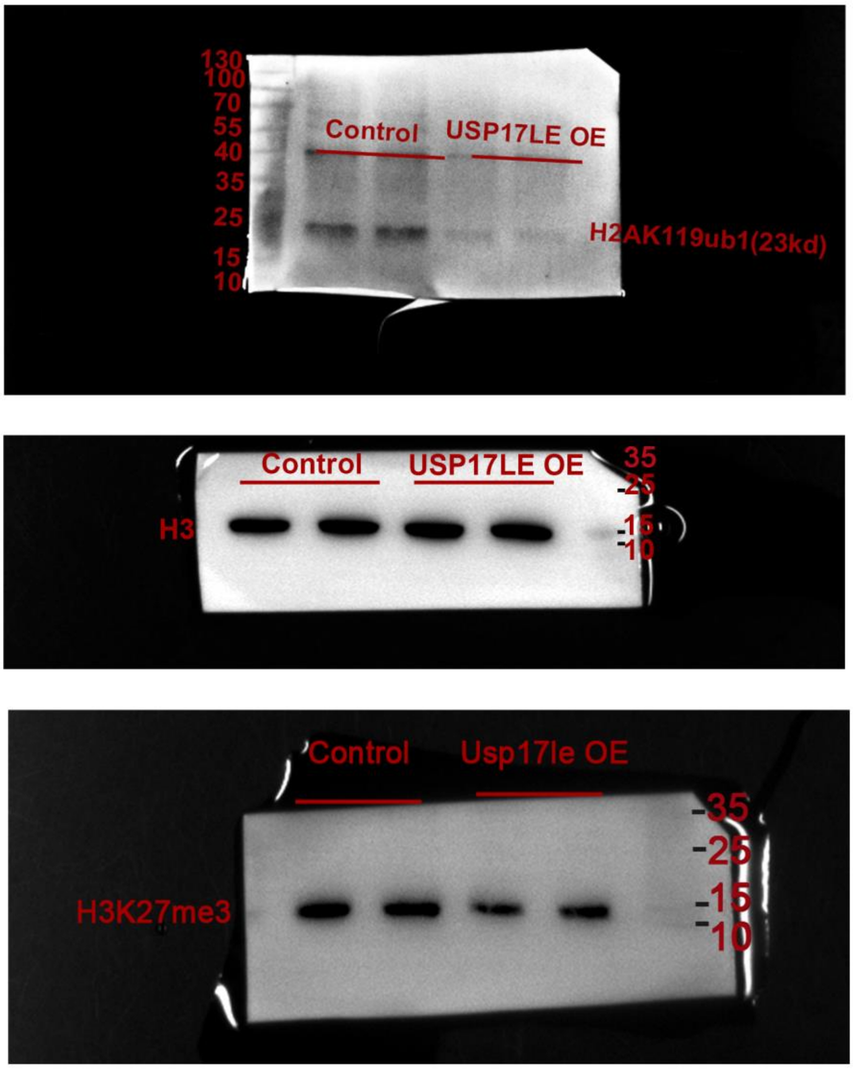

Figure 3F. H2AK119ub1 was detected in Zscan4+ and Zscan4- cells

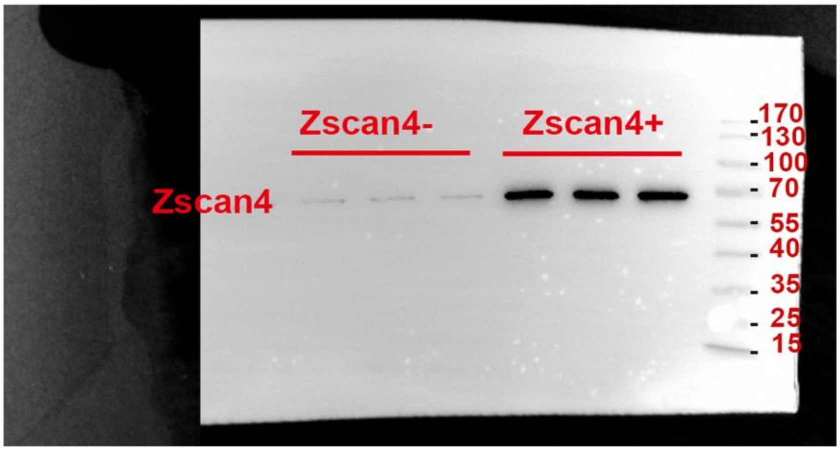

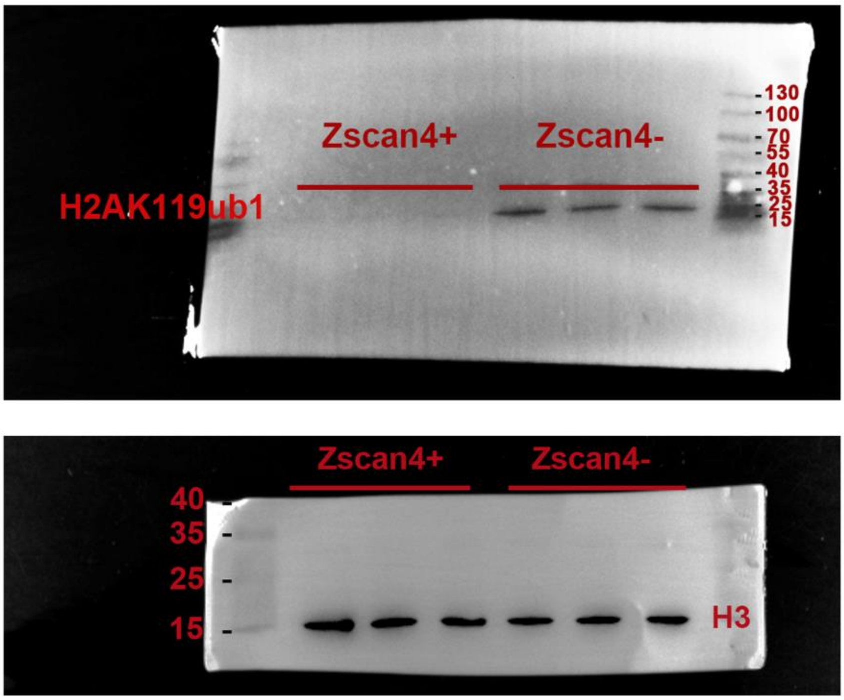

Figure 3H. PRT4165 can rescue Zscan4 in Usp17l KD cell lines

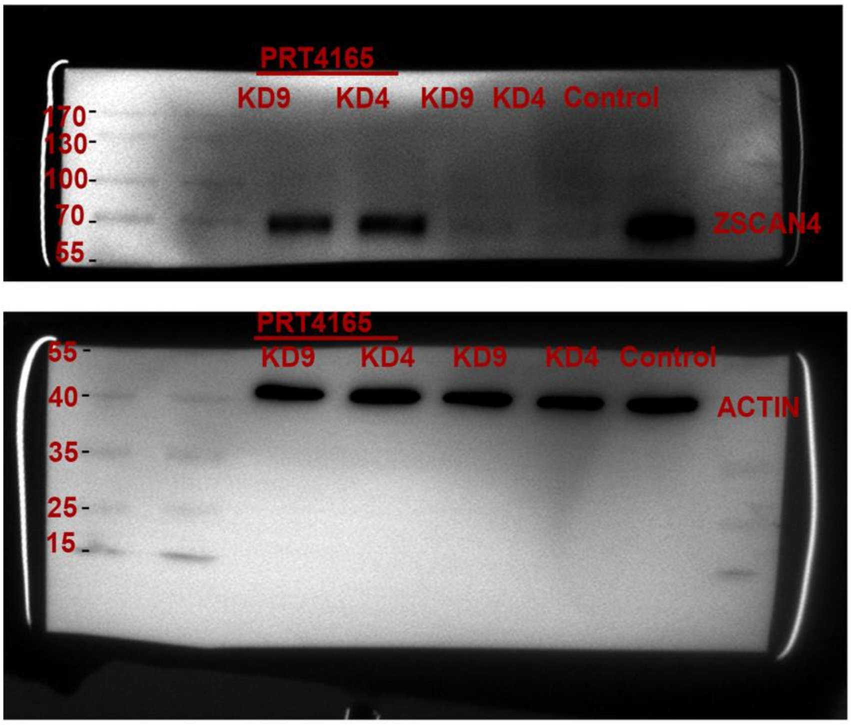

Figure 4A. MG132 prevents degradation of ZSCAN4

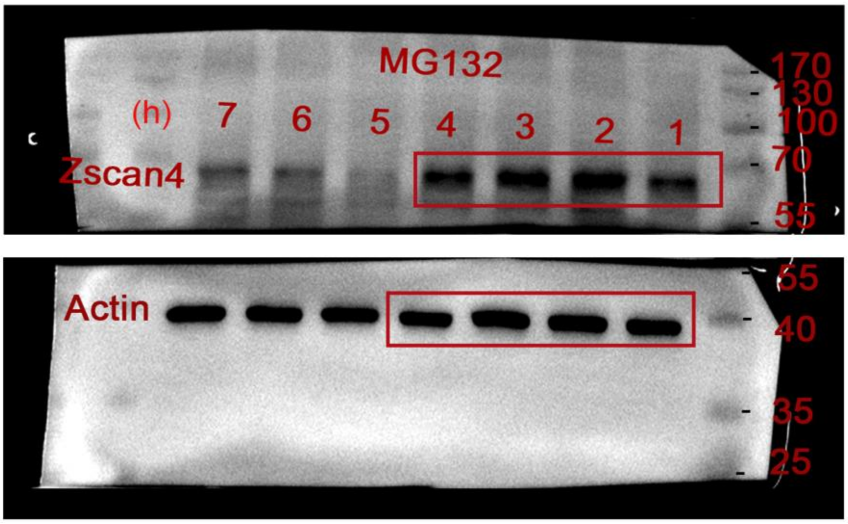

Figure 4B. USP17LE promotes ZSCAN4 expression

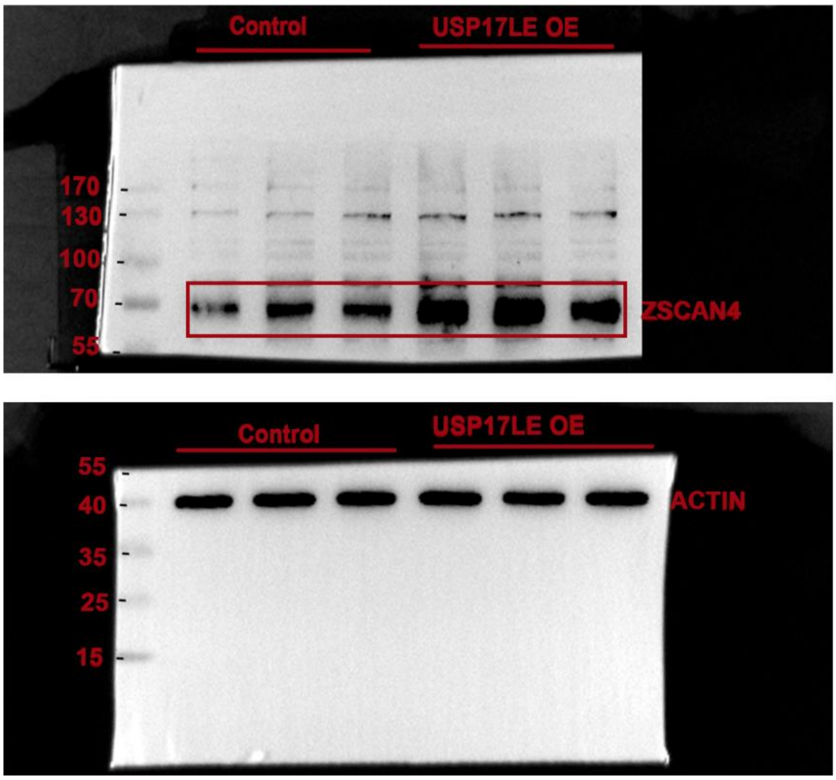

Figure 4D.Overexpression of USP17LE can prevent degradation of ZSCAN4

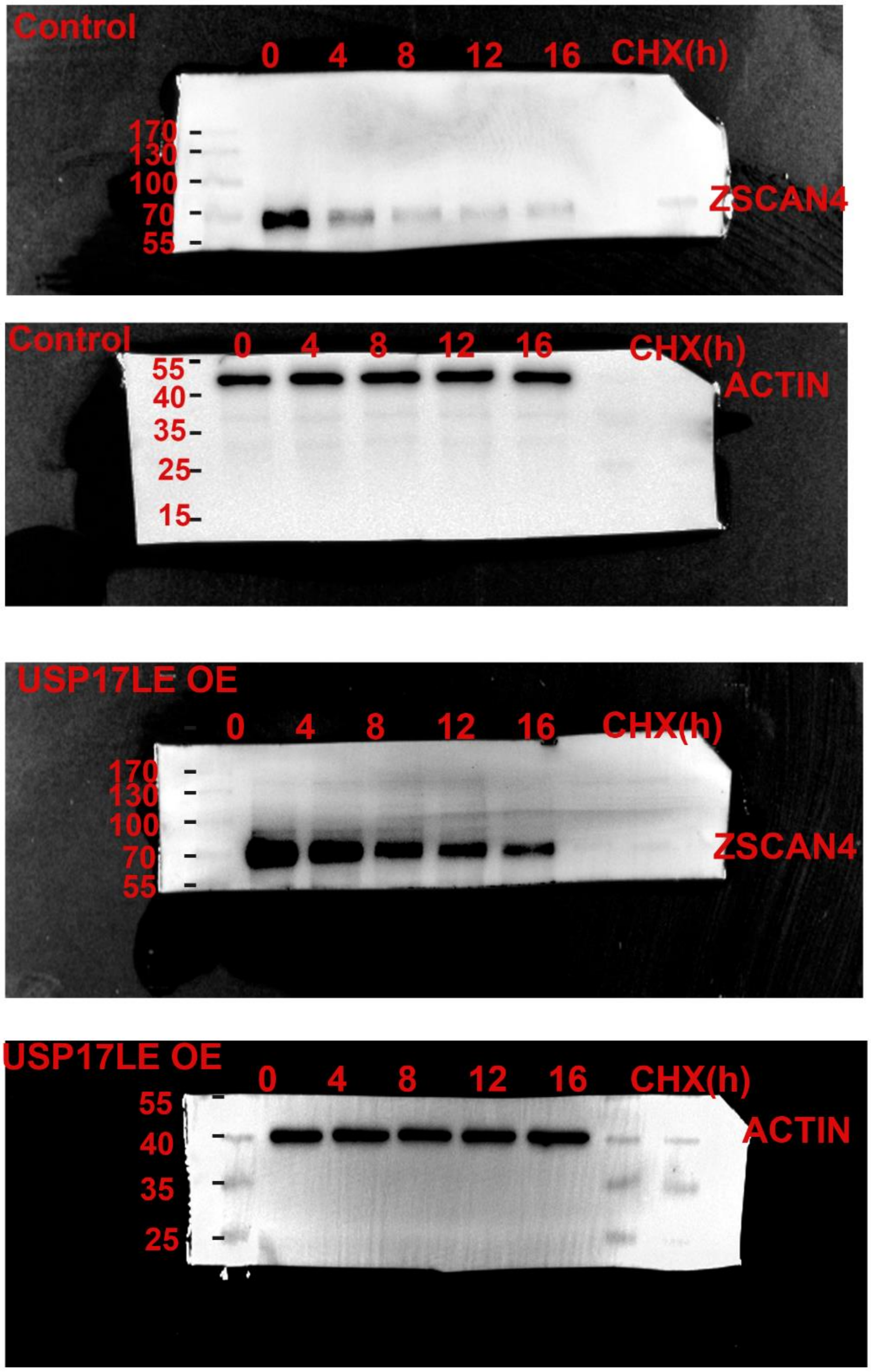

Figure 4E.USP17LE regulates the deubiquitination of ZSCAN4

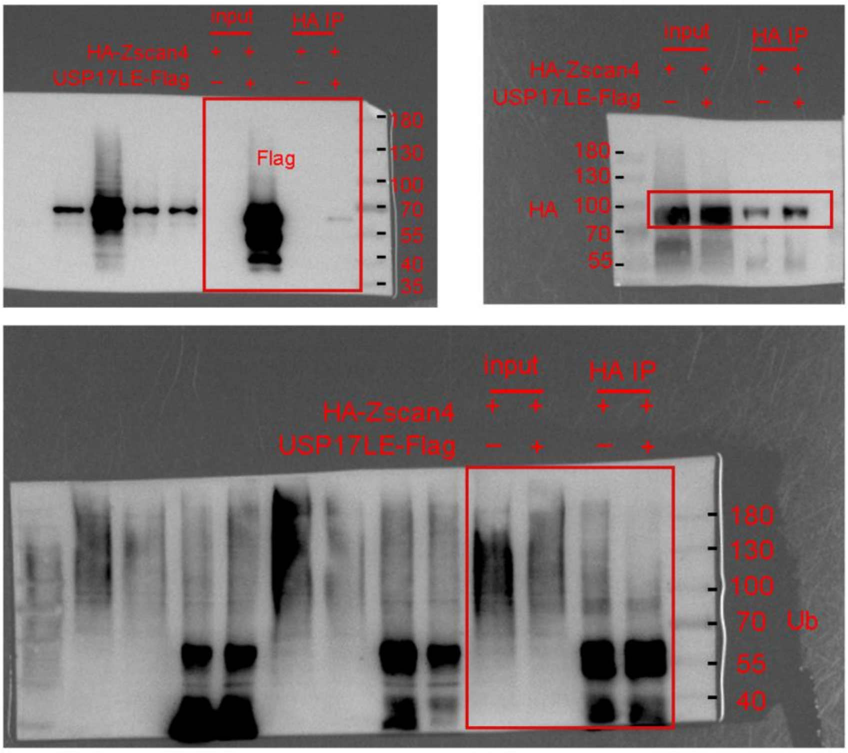

Figure 4F. The protective effect of USP17L on ZSCAN4 disappeared after mutation

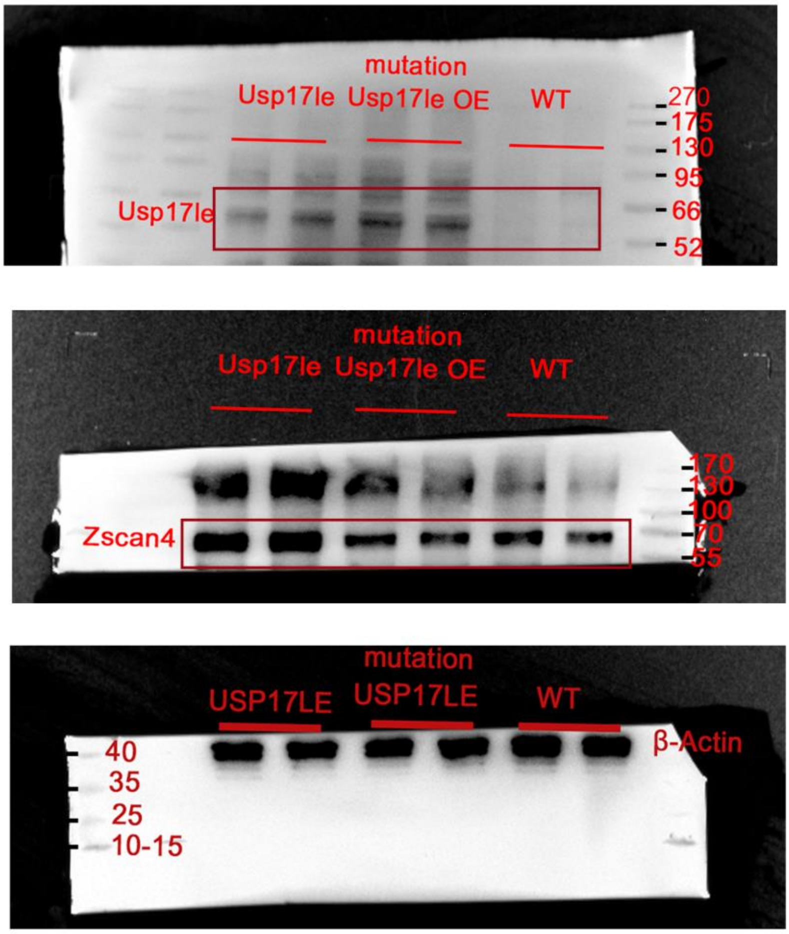

Figure 4H. The USP17L$^{408-506}$ provides no protection to ZSCAN4

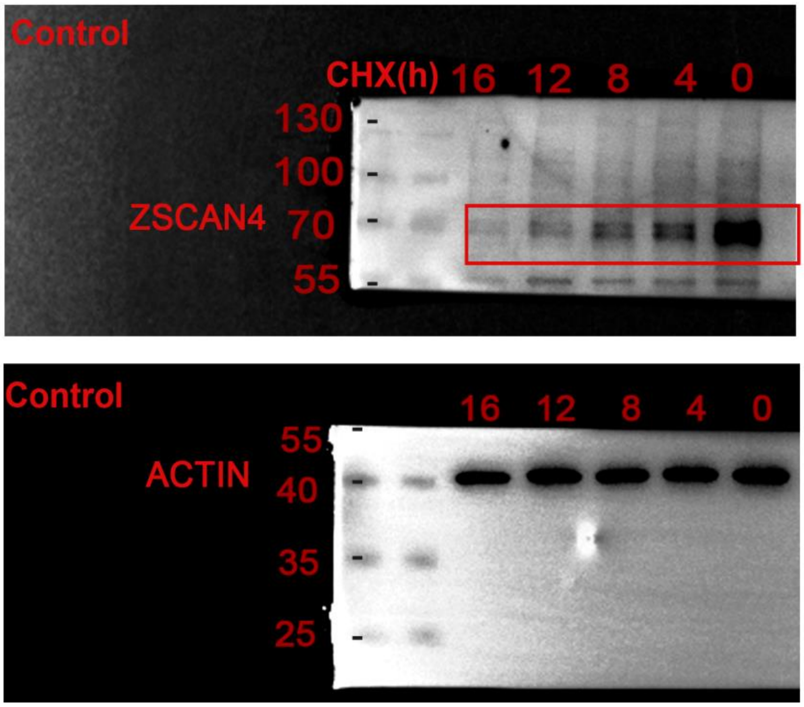

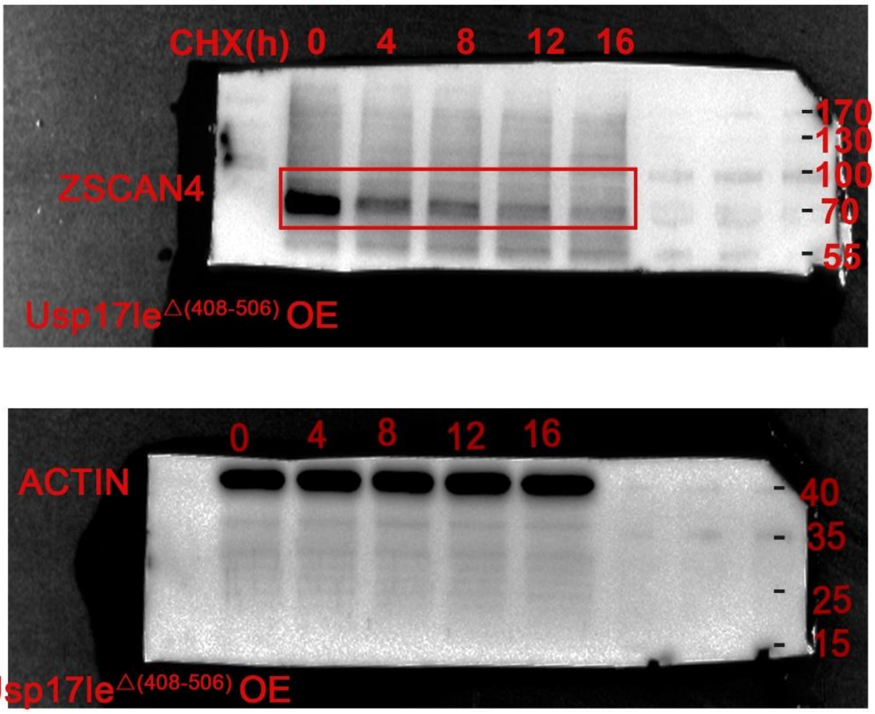

Figure 4J. USP17LE$^{D453A/D457A}$provides no protection to ZSCAN4

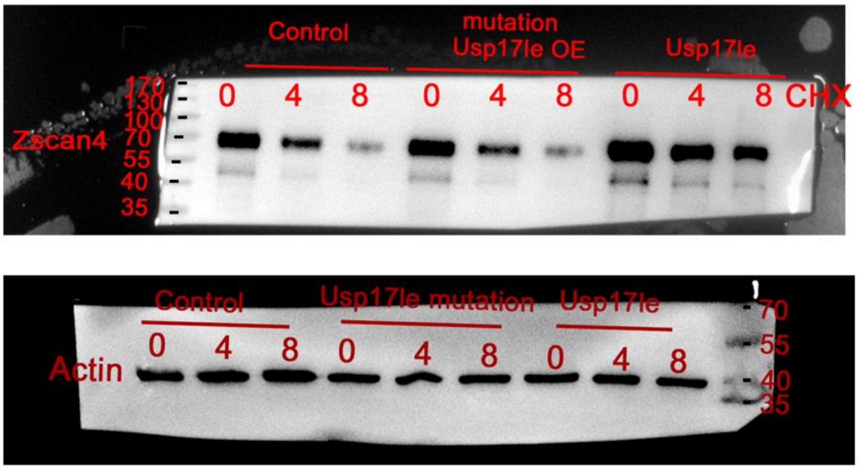

Figure 4K. The overexpression of Usp17l408-506 had no effect on ZSCAN4

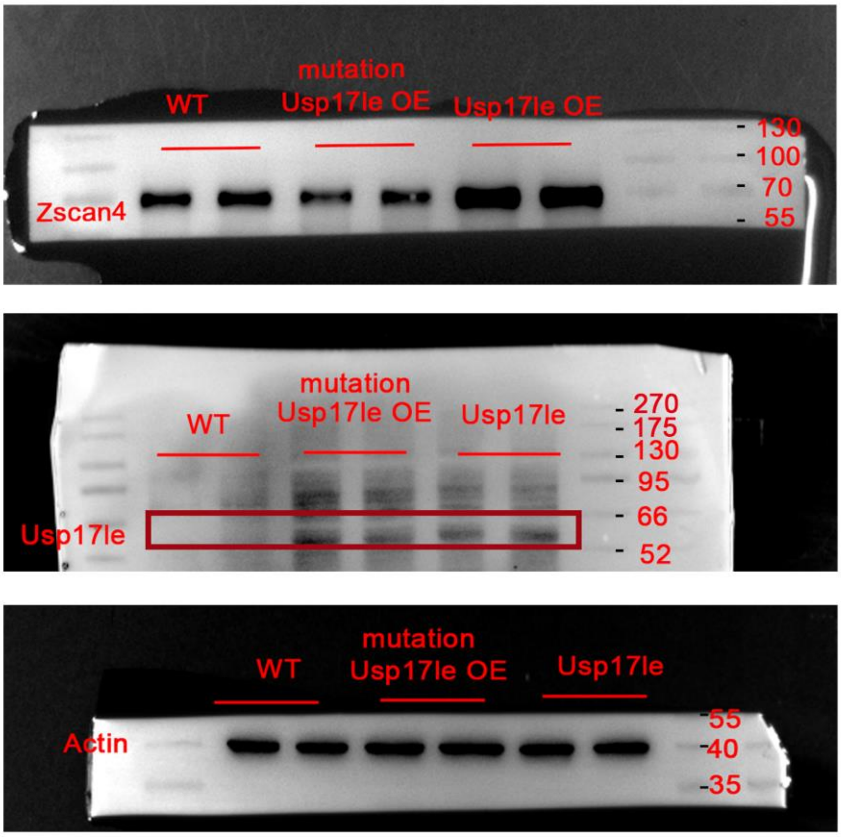

Figure S1B. USP17L was highly expressed in Zscan4+ cells

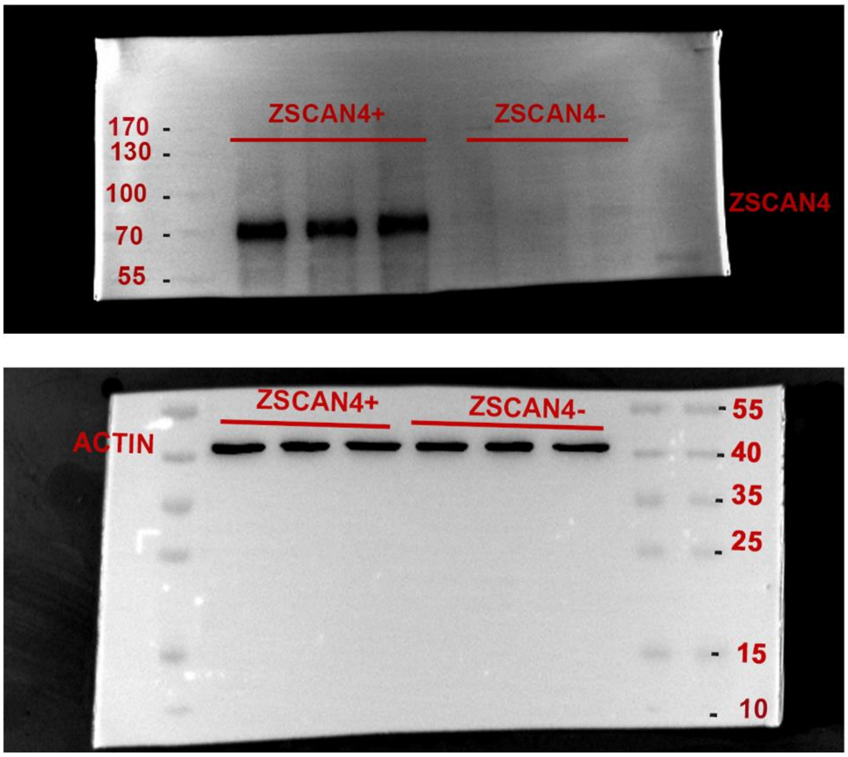

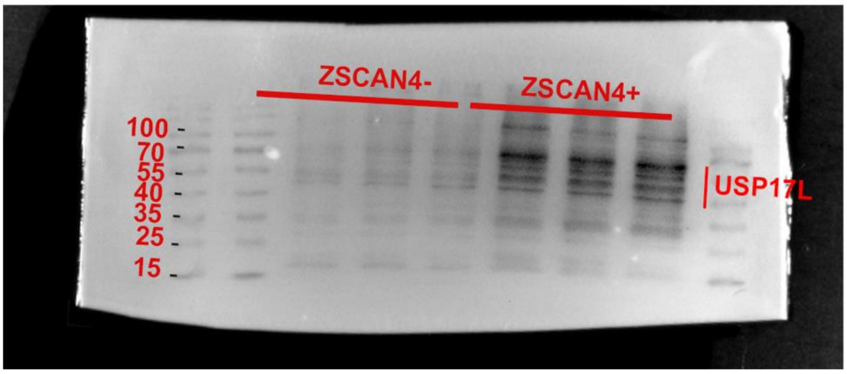

Figure S2A. **Pluripotency gene increased after USP17L knockdown**

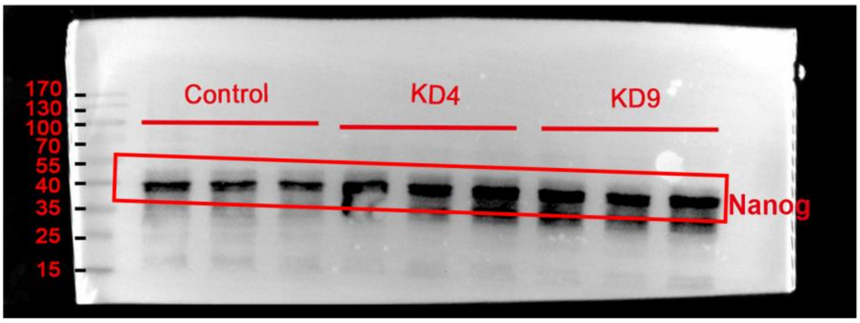

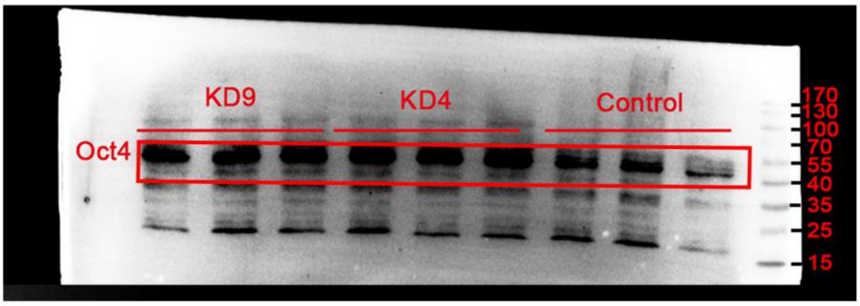

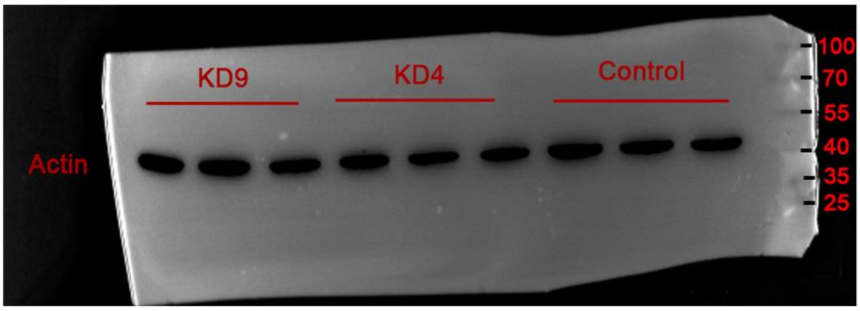

Figure S4C. PRT4165 can reduce H2AK119ub1 level

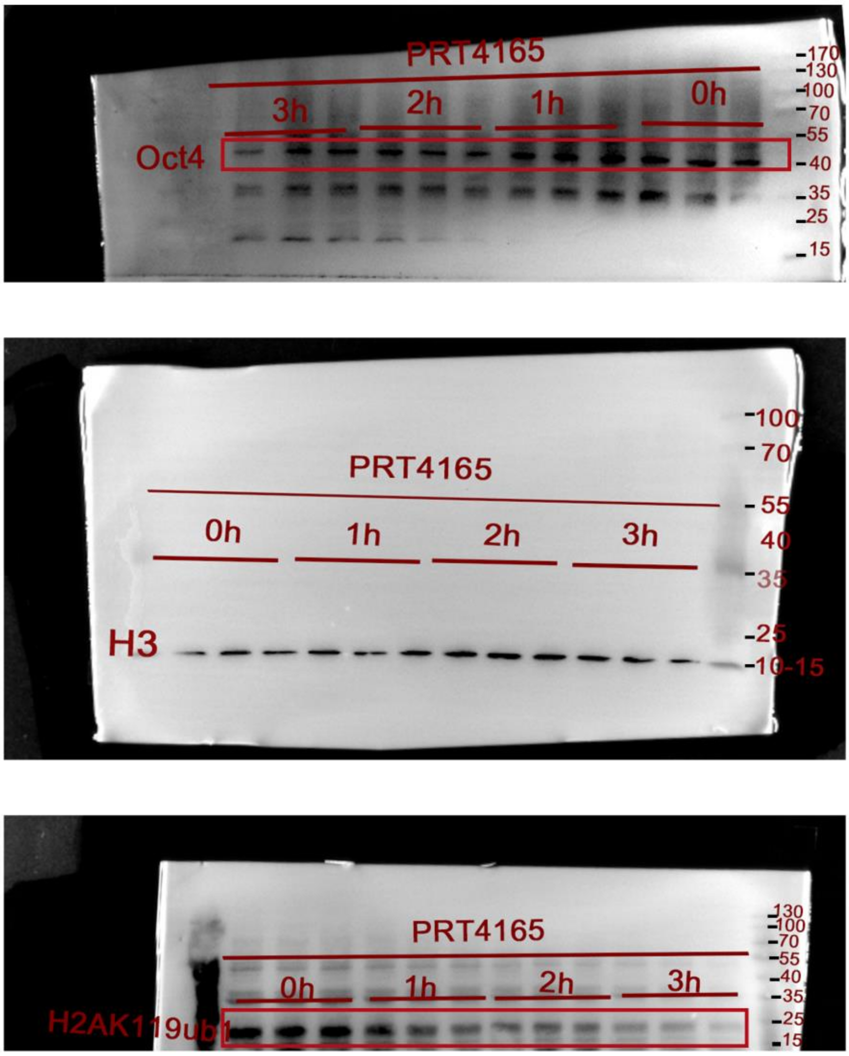

Figure S4E. ZSCAN4 is upregulated after ESCs treatment with PRT4165

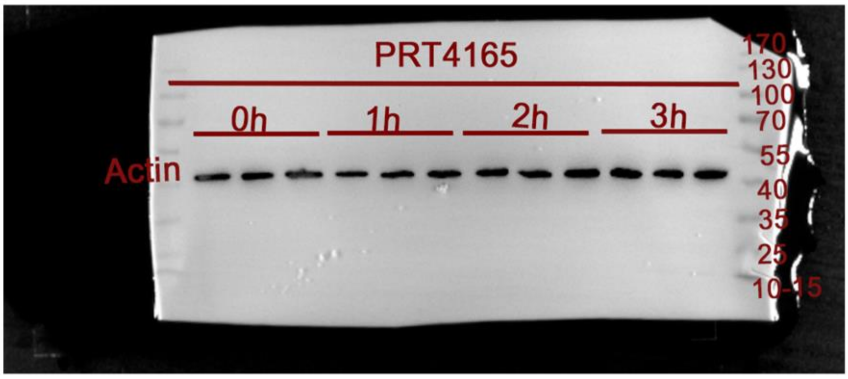

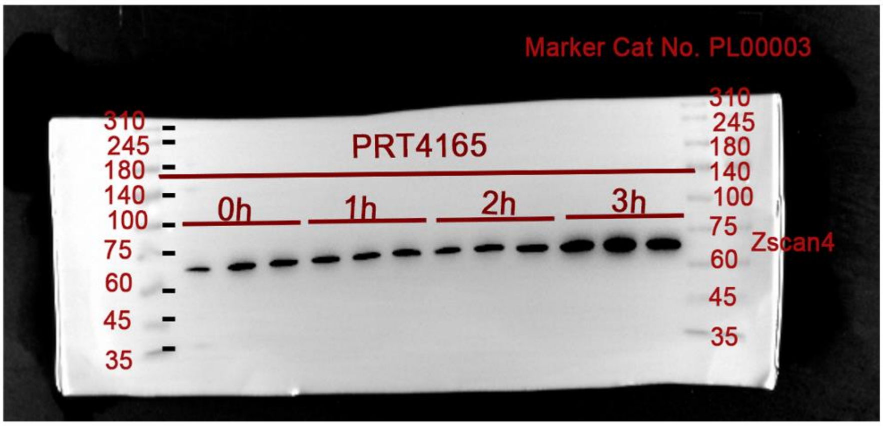

Figure S5A: Changes of Zscan4 after overexpression of Usp17l

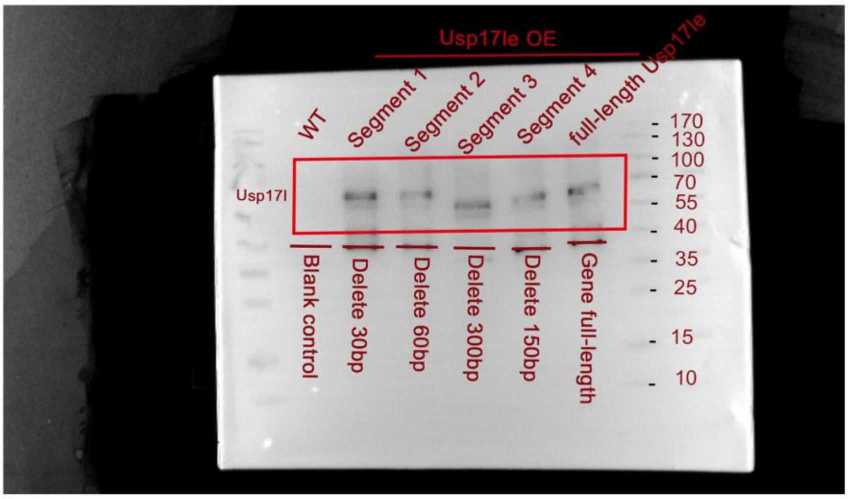

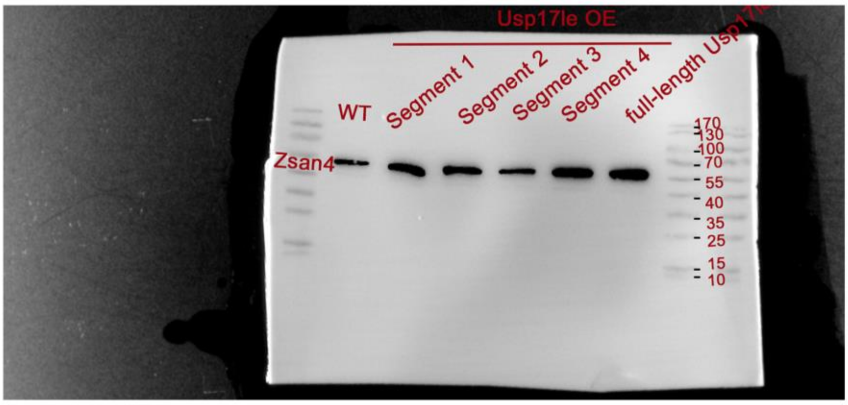

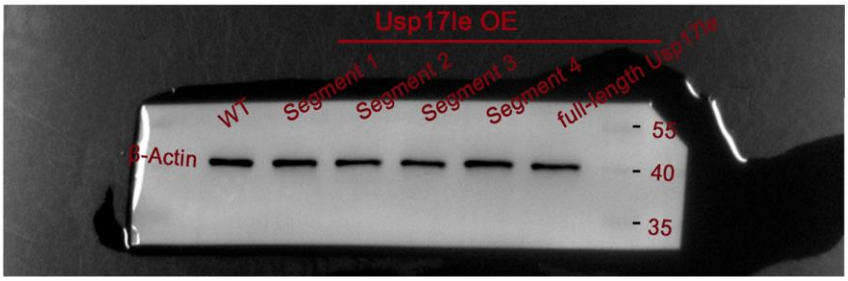

Figure S5B. USP17L$^{D453A/D457A}$ does not interact with ZSCAN4

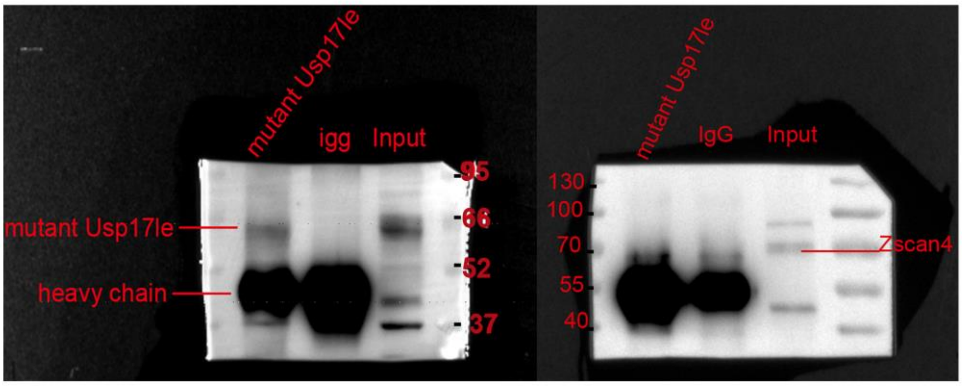

Supplement: Supplementary file 5 — Source Data 2 [file 41467_2025_62303_MOESM5_ESM.pdf]
